# Bone mineral density in women with functional hypothalamic amenorrhea compared to eumenorrheic controls and recently menopausal women

**DOI:** 10.3389/fendo.2026.1897514

**Published:** 2026-08-04

**Authors:** Nancy Safwan, Regina Castaneda, Erin R. Uddenberg, Maria D. Hurtado Andrade, Jenna Maughan, Mariam Saadedine, Stephanie S. Faubion, Sarah L. Berga, Margareta D. Pisarska, C. Noel Bairey Merz, Chrisandra L. Shufelt

**Affiliations:** 1 Division of General Internal Medicine, Mayo Clinic, Jacksonville, FL, United States; 2 Mayo Clinic Center for Women’s Health, Mayo Clinic, Rochester, MN, United States; 3 Division of Endocrinology, Diabetes, Metabolism, and Nutrition, Mayo Clinic, Jacksonville, FL, United States; 4 Precision Medicine for Obesity Program, Mayo Clinic, Rochester, MN, United States; 5 Biostatistics and Bioinformatics Research Center, Cedars-Sinai Medical Center, Los Angeles, CA, United States; 6 Department of Obstetrics and Gynecology, George Washington University, Washington, DC, United States; 7 Department of Obstetrics and Gynecology, Jacobs School of Medicine and Biomedical Sciences, University at Buffalo, Buffalo, NY, United States; 8 Division of Reproductive Endocrinology and Infertility, Department of Obstetrics and Gynecology, Cedars-Sinai Medical Center, Los Angeles, CA, United States; 9 Barbra Streisand Women’s Heart Center, Cedars-Sinai Smidt Heart Institute, Los Angeles, CA, United States

**Keywords:** bone, bone mineral density, functional hypothalamic amenorrhea, hypoestrogenism, menopause, oral contraceptives

## Abstract

**Introduction:**

Functional hypothalamic amenorrhea (FHA) accounts for 30% of secondary amenorrhea and is characterized by hypothalamic hypercortisolemia, hypothalamic hypothyroidism, and anovulation with hypoestrogenemia due to disrupted GnRH drive. While FHA is associated with decreased bone mineralization; it remains unclear how bone health compares to menopausal women, who also experience bone loss. This study compared dual-energy x-ray absorptiometry (DXA) bone health parameters among women with FHA, eumenorrheic controls, and recently menopausal women. It also examined the impact of prior combined oral contraceptive (COC) use on bone mineral density (BMD) in women with FHA.

**Methods:**

This cross-sectional study included 20 women with FHA, 9 eumenorrheic controls, and 12 recently menopausal women who underwent hip and spine DXA. None of the participants were taking hormone therapy. FHA was defined as ≥3 months of amenorrhea, estradiol <50 pg/ml, FSH and LH <10 mIU/L, excluding other etiologies. DXA bone parameters were measured using Lunar iDXA (GE) including BMD from lumbar spine (L1-L4) and hip.

**Results:**

By design, age differed significantly across groups (p < 0.0001), with median ages of 27.8 years [23.4, 34.1] in women with FHA, 29.6 years [28.3, 32.4] in eumenorrheic controls, and 55.3 years [53.5, 56.6] in recently menopausal women. There were no significant differences in weight or BMI across groups. Significant differences were observed at all measured skeletal sites, including all lumbar spine levels, femur neck, total femur, and total BMD (all p < 0.05). Pairwise comparisons revealed that lumbar spine BMD was significantly lower in FHA compared to eumenorrheic controls (p = 0.04), while FHA and menopausal women did not significantly differ from each other (p = 0.07). Among FHA women with prior COC use, L1–4 BMD was not significantly different from controls (p = 0.19).

**Conclusions:**

Lumbar spine BMD in young women with FHA was comparable to that of recently menopausal women, highlighting the profound skeletal impact of chronic estrogen deficiency. Prior COC use may partially attenuate FHA-associated bone loss, though larger studies are needed to confirm this observation. Future studies should explore whether skeletal outcomes vary by FHA etiology and the potential role of COC duration in preserving BMD.

## 1 Introduction

**Figure 1:**
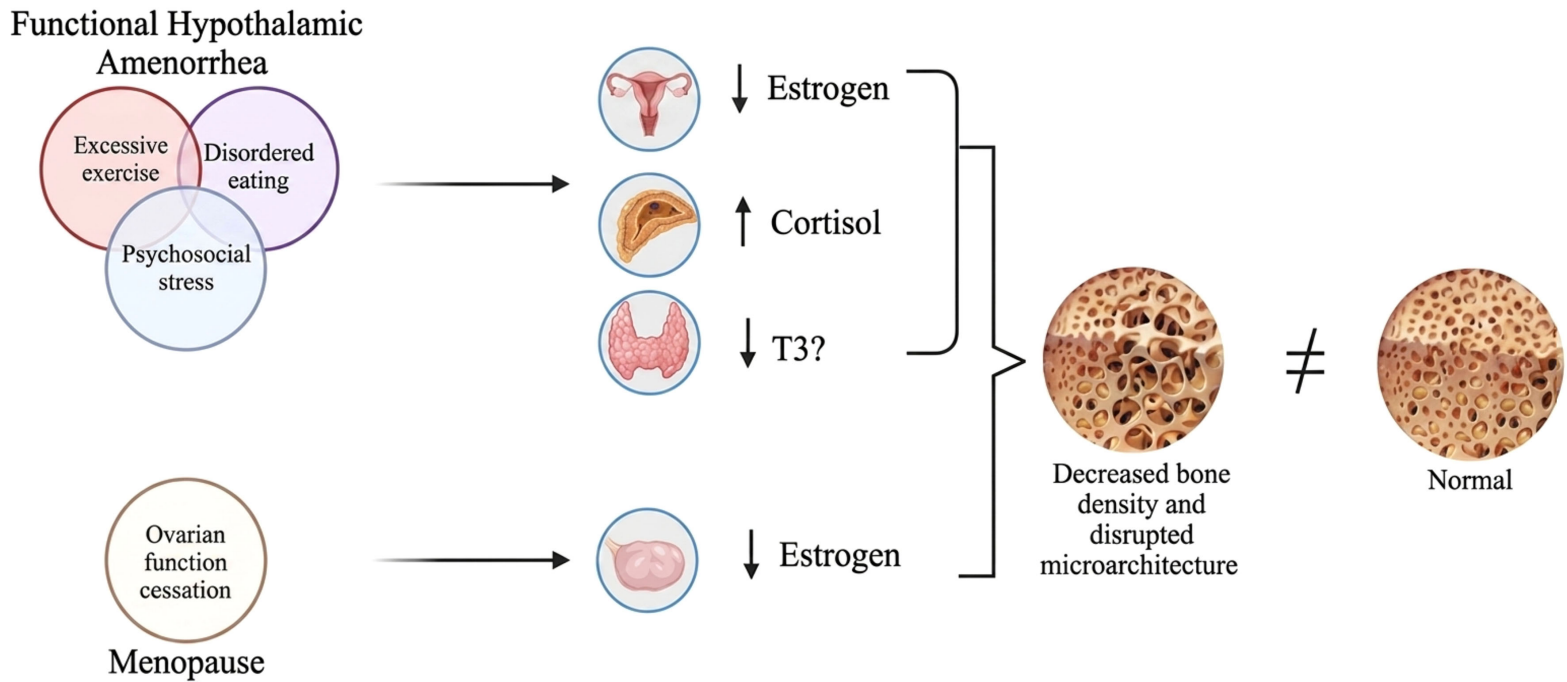
Hormonal drivers of bone loss in functional hypothalamic amenorrhea. Hormonal patterns in functional hypothalamic amenorrhea and menopause contributing to disrupted bone microarchitecture. T3 denotes triiodothyronine.

Functional hypothalamic amenorrhea (FHA) is an anovulatory form of hypogonadotropic hypogonadism that accounts for approximately 30 to 35% of secondary amenorrhea cases (1, 2). It is characterized by a disrupted pulsatile gonadotropin-releasing hormone (GnRH) secretion, resulting in hypoestrogenemia, hypercortisolemia, and hypothyroidism (1, 3). The disruption of the hypothalamic-pituitary axis in women with FHA is typically driven by a combination of factors including psychosocial stress, excessive exercise, and/or disordered/restrictive eating behaviors (2). As a complex neuroendocrine disorder, FHA has significant long-term health consequences including cardiovascular disease, infertility, metabolic disturbances, and disrupted bone microarchitecture (4–6). Regardless of the underlying cause, the chronic hypoestrogenic state associated with FHA can adversely affect bone health (**Figure 1**) (1).

Adolescence and early adulthood are critical windows for bone mass acquisition, with nearly 45–50% of peak bone mass attained during puberty (7, 8). Peak bone mineral density is typically reached between 19–21 years in females and 21–24 years in males, and differs across skeletal sites (9). Estrogen is essential for optimal skeletal development, exerting antiresorptive effects through inhibition of osteoclast activity, promotion of osteoblast survival, stimulation of growth factors, and modulation of inflammatory cytokines and vitamin D receptor expression (10–12). Consequently, sustained hypoestrogenemia in young women is associated with increased bone turnover, decreased bone mineral density (BMD), and increased risk of osteoporosis (13–15).

This study aimed to compare the BMD in young women with FHA, eumenorrheic controls not currently using hormonal contraceptives, and recently menopausal women (<3 years) not receiving menopausal hormone therapy. We hypothesized that premenopausal women with FHA have lower BMD than both eumenorrheic controls and recently menopausal women.

## 2 Materials and methods

### 2.1 Data source and study participants

Participants were recruited through obstetrics and gynecology referrals, social media advertisements, and review of electronic health records in the Los Angeles area between 2017 and 2021. All participants provided informed consent and agreed to the use of their data for research purposes. The study was approved by Cedars-Sinai Medical Center Institutional Review Board (IRB).

Women were considered to have FHA if they experienced the absence of menstrual periods for at least three consecutive months and met the following hormonal criteria: estradiol (E2) <50 pg/mL, follicle-stimulating hormone (FSH) and luteinizing hormone (LH) levels <10 mIU/mL, and an LH: FSH ratio <1. A follow-up E2 measurement <50 pg/mL one week later was required to confirm the diagnosis. Other causes of secondary amenorrhea, such as premature ovarian failure, thyroid disorders, hyperprolactinemia, pregnancy, and PCOS, were excluded. Eumenorrheic controls were aged 18–40 years, reported regular monthly menstrual cycles, and had confirmed ovulation by a day 22–24 progesterone level >3 ng/mL. Recently menopausal women were over 50 years old, had experienced natural menopause within the past 3 years (defined as 12 months of amenorrhea not medically induced by surgery or chemotherapy), and had confirmatory FSH (>30 IU/L) and E2 (<10 pg/mL) levels.

Exclusion criteria for all participants included recognized cardiovascular risk factors (e.g., smoking, hypertension, hyperlipidemia, or diabetes); known thyroid disease or thyroid dysfunction; current use of hormone therapy or combined oral contraceptives (COCs); use of medications or substances that could contribute to amenorrhea; major Axis I disorders other than depression; pregnancy within the past 12 months; or breastfeeding within the past 6 months.

### 2.2 Data collection

Demographic information, self-administered questionnaires, anthropometric measures (height and weight), and fasting blood levels of reproductive and metabolic hormones were collected at baseline. Reproductive and lifestyle questionnaires were used to collect information on age at menarche, date of last menstrual cycle, prior use of COC or hormone therapy, and exercise patterns, including self-reported duration, intensity, and weekly frequency. Baseline blood draws were performed after an overnight fast, and hormonal assay were conducted on stored serum samples at the Hormone Core Laboratory, University of Southern California, Los Angeles, California. Samples were analyzed in duplicate. Dual-energy x-ray absorptiometry (DXA) was performed using Lunar iDXA (GE) to assess skeletal status. All examinations were performed by the same trained technician and following standard positioning protocols. The scanner underwent daily calibration using the manufacturer’s quality assurance phantom in accordance with institutional quality assurance procedures. Bone mineral density (BMD) was reported as absolute values (g/cm²), as this metric allows direct comparison across the study groups without reliance on age- or peak bone mass–referenced T-scores or age-adjusted Z-scores, which are not directly comparable across heterogeneous reproductive stages. Height (cm) and weight (kg) were measured on the day of the scan, and BMI (kg/m²) was calculated.

### 2.3 Statistical analyses

Continuous variables are presented as median [interquartile range] and compared using the Kruskal-Wallis test. Categorical variables are presented as count (%) and compared using Fisher’s exact test. Pairwise comparisons were subsequently performed using Mann-Whitney U test. Correlation analyses were conducted among women with FHA using Spearman rank correlation coefficients (ρ) to assess associations between BMD, reproductive hormones, duration of amenorrhea, and COC exposure, including duration of use. All analyses were conducted using BlueSky Statistics (version 10.3.7). Figures were created using GraphPad Prism (version 10.4.2). Statistical significance was set at p < 0.05 for all analyses.

## 3 Results

### 3.1 Baseline characteristics

A total of 41 participants were included and categorized into three groups: FHA (n=20), eumenorrheic controls (n=9), and recently menopausal women (n=12) (**Table 1**). The groups differed significantly in age (p<0.0001), with women with FHA being the youngest (median 27.8 [23.4, 34.1] years). There were no significant group differences in weight, BMI, COC use, or age at menarche. As expected, the number of months since last menstrual period (LMP) was significantly greater in FHA compared to eumenorrheic controls and menopausal women (p <0.0001). As expected by study design, E2, LH, FSH, and the LH: FSH ratio differed significantly across groups (all p < 0.01). Women with FHA demonstrated hypoestrogenemia and suppressed gonadotropins compared with eumenorrheic controls, while recently menopausal women exhibited markedly elevated gonadotropins and low estradiol, confirming postmenopausal status. Thyroid-stimulating hormone (TSH) did not differ significantly among groups (p = 0.54); however, triiodothyronine (T3) and thyroxine (T4) concentrations were significantly lower in women with FHA compared with both eumenorrheic controls and recently menopausal women (both p < 0.01), consistent with the hypothalamic hypothyroidism previously described in FHA. No participant was receiving osteoporosis-specific therapy (e.g., bisphosphonates, denosumab, teriparatide, or selective estrogen receptor modulators) or medications known to affect bone metabolism (e.g., systemic glucocorticoids, antiepileptic drugs, aromatase inhibitors, gonadotropin-releasing hormone agonists, or heparin). The absence of osteoporosis-specific therapy was expected, as the postmenopausal cohort comprised recently menopausal women.

**Table 1:** Clinical characteristics of participants by group.

Variable	FHA (n=20)	Controls (n=9)	Recently menopausal (n=12)	p-value
Age (yrs)	27.8 [23.4, 34.1]	29.6 [28.3, 32.4]	55.3 [53.5, 56.6]	**<0.0001**
Race, White (%)	18 (90%)	4 (44%)	6 (50%)	**0.024**
Weight (kgs)	57.4 [49.9, 66.2]	57.6 [54.4, 71.2]	53.3 [48.7, 56.0]	0.257
BMI (kg/m^2^)	21.1 [19.5, 23.1]	21.3 [20.0, 24.0]	19.7 [18.7, 20.3]	0.727
Alcohol use, yes	2 (10%)	2 (22%)	3 (25%)	0.496
Moderate-to-vigorous physical activity, hours/week	5.7 [3.7, 7.2]	4.0 [3.0, 5.0]	4.5 [3.3, 5.1]	0.287
Prior COC use, yes (%)	15 (75%)	4 (44%)	5 (45%)	0.162
Number of months since LMP	26 [5, 46.5]	--	22 [16.5, 34]	**<0.0001**
Age at menarche (yrs)	13 [12, 14.5]	12 [11, 12.5]	12.5 [12, 13]	0.061
E2 (pg/mL)	28.5 [24.0, 40.5]	47.6 [37.0, 65.5]	5.8 [0.0, 8.5]	**<0.001**
LH (mIU/mL)	2.4 [0.0, 3.3]	6.6 [6.1, 7.7]	44.1 [35.2, 48.1]	**<0.0001**
FSH (mIU/mL)	5.8 [4.9, 7.1]	6.4 [4.9, 7.8]	71.2 [0.0, 85.2]	**<0.0001**
LH: FSH ratio	0.3 [0.0, 0.6]	1.1 [0.5, 1.3]	0.6 [0.5, 0.8]	**<0.01**
TSH (μU/mL)	1.6 [1.0, 2.1]	1.8 [1.4, 2.1]	1.8 [1.5, 2.2]	0.54
T3 (μg/dL)	84.5 [79.8, 95.8]	111.0 [105.0, 119.0]	105.0 [92.5, 113.0]	**<0.01**
T4 (ng/dL)	6.0 [5.5, 6.6]	7.2 [6.9, 8.0]	6.8 [6.6, 7.5]	**<0.01**

Data are presented as median [interquartile range] for continuous variables and as n (%) for categorical variables. Group comparisons performed using Kruskal-Wallis test for non-normally distributed variables, and Fisher’s exact test for categorical variables. BMI, body mass index; COC, combined oral contraceptive; E2, estradiol; FHA, functional hypothalamic amenorrhea; FSH, follicle-stimulating hormone; LH, luteinizing hormone; LH, FSH ratio, luteinizing hormone-to-follicle-stimulating hormone ratio; LMP, last menstrual period; T3, triiodothyronine; T4, thyroxine; TSH, thyroid-stimulating hormone. Values shown in bold indicate statistical significance (p < 0.05).

### 3.2 Bone mineral density

**Figure 2:**
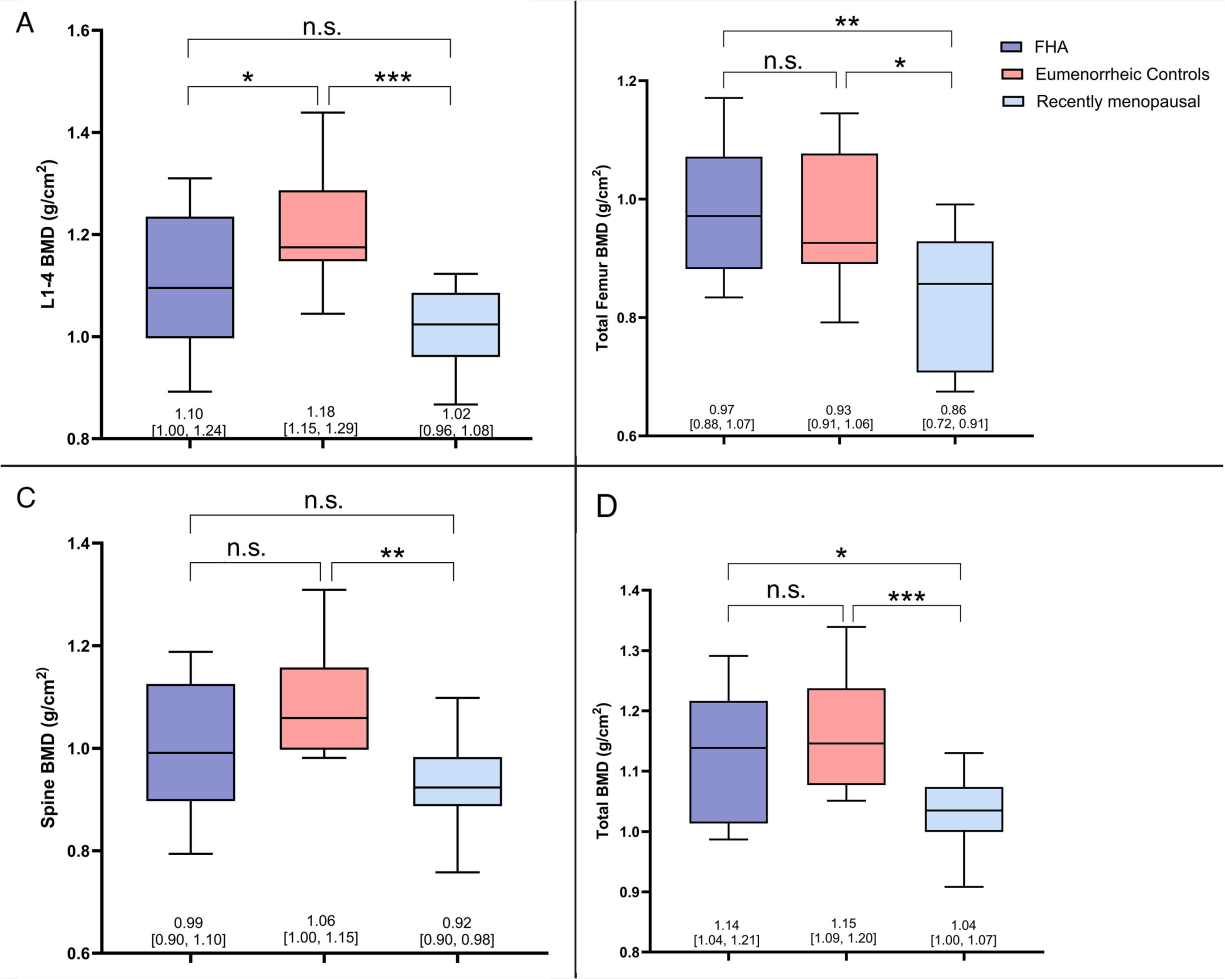
Bone mineral density at lumbar spine and femoral sites across women with functional hypothalamic amenorrhea, eumenorrheic controls, and recently menopausal women. Data presented as median [interquartile range]. Group comparisons were performed using Kruskal-Wallis test with pairwise post hoc comparisons. **(A)** L1–4 lumbar spine BMD (FHA vs. controls, p = 0.04; FHA vs. menopausal, p = 0.07; controls vs. menopausal, p < 0.001); **(B)** Total femur BMD (FHA vs. controls, p = 0.94; FHA vs. menopausal, p < 0.01; controls vs. menopausal, p = 0.02); **(C)** Composite spine BMD (FHA vs. controls, p = 0.08; FHA vs. menopausal, p = 0.15; controls vs. menopausal, p < 0.01); **(D)** Total BMD (FHA vs. controls, p = 0.45; FHA vs. menopausal, p = 0.02; controls vs. menopausal, p < 0.001). Statistical significance is indicated as *p < 0.05,** p < 0.01,*** p < 0.001. BMD, bone mineral density; FHA, functional hypothalamic amenorrhea.

**Figure 3:**
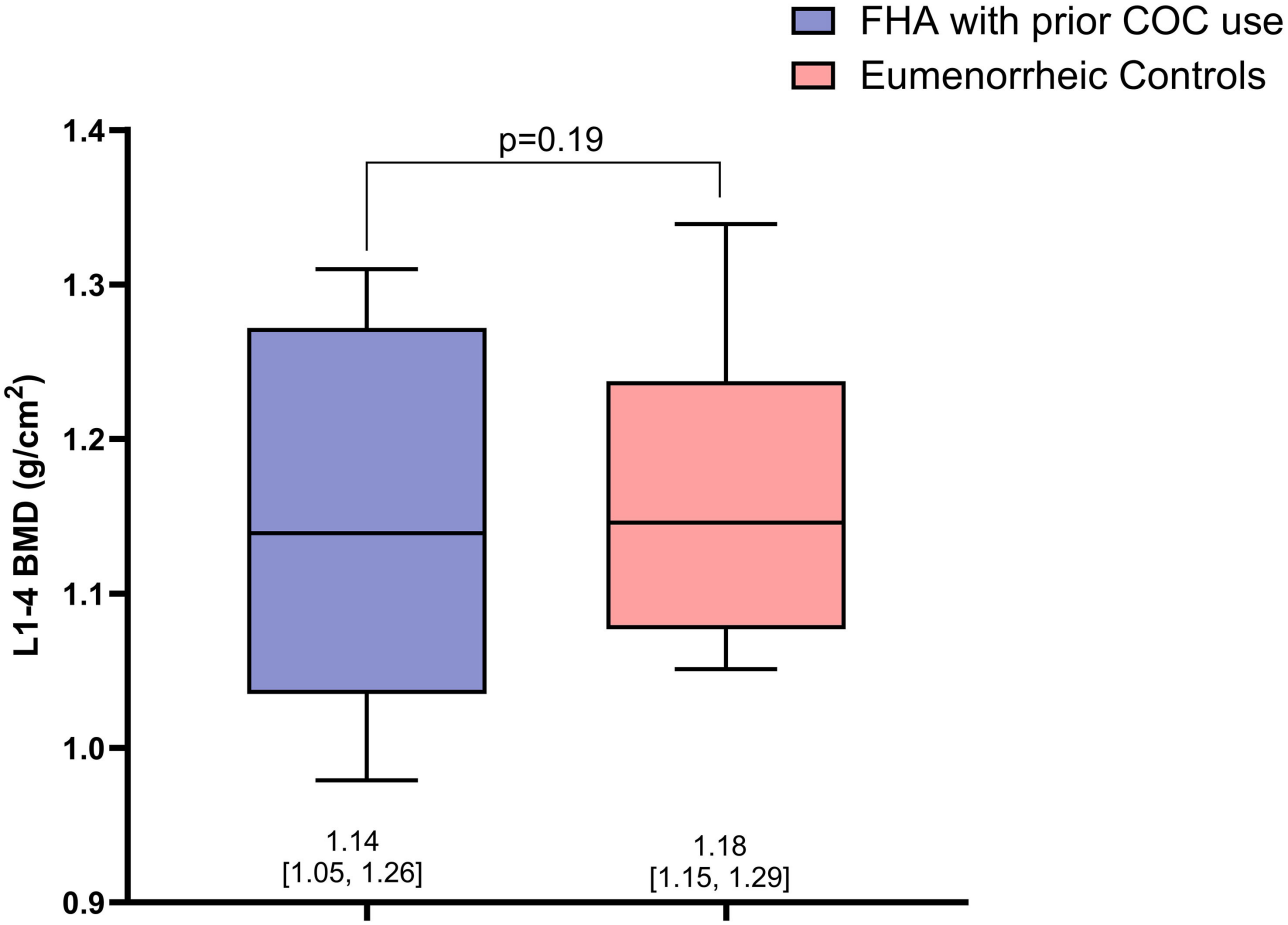
L1–4 bone mineral density levels in women with functional hypothalamic amenorrhea with prior combined oral contraceptive use and eumenorrheic controls. Data presented as median [interquartile range]. Comparison was performed using Mann-Whitney U test between women with FHA who reported prior COC use and eumenorrheic controls (p = 0.19). COC, combined oral contraceptive; FHA, functional hypothalamic amenorrhea; BMD, bone mineral density.

Significant differences across the three groups were observed at all lumbar spine sites, including L1-4, L2-4, and individual vertebral levels L1 through L4, as well as at the femoral neck, total femur, composite spine and total BMD (**Table 2**). *Post hoc* pairwise comparisons revealed that for L1–4 BMD, eumenorrheic controls had significantly higher values than women with FHA (p = 0.04), and recently menopausal women had significantly lower values than controls (p < 0.01), while FHA and menopausal groups did not differ significantly from each other (p = 0.07; **Figure 2A**). A similar pattern was observed for spine BMD, where FHA values trended lower than controls without reaching significance (p = 0.08), and did not differ from the menopausal group (p = 0.15; **Figure 2C**). In contrast, at femoral and total skeletal sites, FHA participants did not differ significantly from eumenorrheic controls (total femur p = 0.94; total BMD p = 0.45), but had significantly higher values than recently menopausal women (total femur p < 0.01; total BMD p = 0.02; **Figures 2B, D**). Among women with FHA who reported prior COC use, L1–4 BMD was not significantly different from eumenorrheic controls (median 1.14 [1.05, 1.26] vs. 1.18 [1.15, 1.29] g/cm²; p = 0.19; **Figure 3**).

**Table 2:** Bone mineral density measures by site and group.

Variable	FHA (n=20)	Controls (n=9)	Recently menopausal (n=12)	p-value
L1–4 BMD (g/cm2)	1.10 [1.00, 1.24]	1.18 [1.15, 1.29]	1.02 [0.96, 1.08]	**0.0022**
L2–4 BMD (g/cm2)	1.12 [1.02, 1.27]	1.20 [1.18, 1.32]	1.06 [0.98, 1.12]	**0.0032**
L1 BMD (g/cm2)	0.99 [0.90, 1.11]	1.1 [1.03, 1.18]	0.92 [0.89, 1.01]	**0.0094**
L2 BMD (g/cm2)	1.10 [0.99,1.24]	1.15 [1.14, 1.27]	1.00 [0.94, 1.08]	**0.0023**
L3 BMD (g/cm^2^)	1.15 [1.06, 1.30]	1.26 [1.20, 1.38]	1.07 [1.01, 1.16]	**0.0015**
L4 BMD (g/cm^2^)	1.09 [1.05, 1.26]	1.21 [1.19, 1.34]	1.08 [0.98, 1.11]	**0.0163**
Femur neck (g/cm^2^)	0.96 [0.90, 1.05]	0.96 [0.88, 1.03]	0.80 [0.73, 0.81]	**<0.0001**
Total femur (g/cm^2^)	0.97 [0.88, 1.07]	0.93 [0.91, 1.06]	0.86 [0.72, 0.91]	**0.0083**
Spine BMD (g/cm^2^)	0.99 [0.90, 1.10]	1.06 [1.00, 1.15]	0.92 [0.90, 0.98]	**0.0162**
Total BMD (g/cm^2^)	1.14 [1.04, 1.21]	1.15 [1.09, 1.20]	1.04 [1.04, 1.07]	**0.0078**

Data presented as median [interquartile range]. Group comparisons were performed using Kruskal-Wallis test. BMD, bone mineral density; FHA, functional hypothalamic amenorrhea. Values shown in bold indicate statistical significance (p < 0.05).

Among women with FHA, there were no significant correlations between BMD at any skeletal site and estradiol, LH, FSH, or the LH: FSH ratio (all |ρ| ≤ 0.32, *p* > 0.05; **Supplementary Figure 1**). In contrast, a greater number of months since the last menstrual period was associated with lower BMD at three of the four skeletal sites, including the lumbar spine (L1–L4) (ρ = −0.56, *p* < 0.05), femoral total BMD (ρ = −0.54, *p* < 0.05), and spine BMD (ρ = −0.56, *p* < 0.01). Although a similar inverse correlation was observed for total BMD, the association did not reach statistical significance (ρ = −0.41, *p* > 0.05). Among women with prior COC exposure, a longer duration of COC use was positively correlated with BMD at all four skeletal sites, with the strongest correlation observed for lumbar spine (L1–L4) BMD (ρ = 0.76, *p* < 0.001), followed by spine BMD (ρ = 0.71, *p* < 0.001), total BMD (ρ = 0.71, *p* < 0.001), and femoral total BMD (ρ = 0.56, *p* < 0.05).

## 4 Discussion

To our knowledge, this study is among the first to comprehensively evaluate the BMD of young women with FHA and directly compare these metrics to both eumenorrheic controls and recently menopausal women. Our findings highlight the significant impact of FHA on bone, revealing that lumbar BMD in young women with FHA is not only reduced compared to their eumenorrheic peers, but is also comparable to that of women who have recently transitioned into menopause. Notably, among women with FHA who had a history of COC use, lumbar spine (L1–L4) BMD did not differ significantly from that of controls, suggesting a potential protective association of COC exposure with bone health. Consistent with this observation, longer duration of COC use was positively correlated with BMD across all skeletal sites. In contrast, BMD inversely correlated with months since last menstrual period at the lumbar spine, femoral, and composite spine sites, suggesting that the cumulative duration of hypothalamic-pituitary-ovarian axis suppression.

Consistent with prior research, women with FHA had significantly lower BMD compared to eumenorrheic controls, particularly in the trabecular-rich regions most sensitive to estrogen deficiency (16). These findings align with prior studies showing hypoestrogenism in FHA contributes to impaired bone accrual and accelerated bone loss (17–20). For instance, a 2-year longitudinal observational study of young women (mean age 22 years) compared bone health of exercising ballet dancers (n=54, FHA = 22) to non-dancer controls (n=57, FHA = 22) to examine the effects of prolonged hypoestrogenism with and without exercise (19). Results showed that women with FHA had a lower bone density that remained below normal controls over two years, independent of age, weight, and exercise (p<0.05) (19). The amenorrheic dancers showed the most stress fractures and the most distorted eating patterns with a BMD lower than that of their normal counterparts. Notably, none of the seven women who resumed menstruation achieved BMD normalization, suggesting that early and sustained hypoestrogenemia may result in a permanent deficit in peak bone mass, highlighting the critical need for timely preventive or therapeutic interventions (19). An important limitation to this study was the large dropout rate (42%), which is not uncommon in young populations (21).

It is important to note that the regional pattern characterized by greater lumbar spine involvement in BMD differences across skeletal sites is biologically plausible, as trabecular bone, which is predominant at the lumbar spine, has higher metabolic turnover and is more responsive to changes in estrogen availability than cortical bone (22, 23). Accordingly, the lumbar spine is often the earliest and most severely affected site in FHA, whereas cortical-rich sites such as the femoral neck and hip tend to be relatively preserved, particularly in earlier stages of disease, although they may also show impairment with prolonged hypoestrogenism.

When comparing women with FHA to recently menopausal women, our findings suggest that mechanisms beyond estrogen deficiency may contribute to the observed bone deficits. Despite being younger and presumably at a stage where bone formation should remain active, women with FHA exhibited BMD comparable to those seen in menopause, a period characterized by accelerated bone loss (24). The complex hormonal disruptions inherent to FHA including hypercortisolemia and hypoestrogenemia, collectively promote a catabolic skeletal environment that may mirror or even exacerbate the bone loss seen in natural menopause (22). In addition, reduced triiodothyronine (T3) availability may contribute to altered bone metabolism, with experimental data suggesting potential effects on osteoblast differentiation and activity, although its overall role in bone health remains less clearly defined (25). Critically, unlike menopause, FHA occurs during the peak bone-building years, amplifying its potential long-term consequences (22).

The severity of BMD decline in FHA may vary by subtype, including energy deficiency–related, exercise-associated, and stress-related presentations, can lead to bone mass that meets clinical thresholds for low BMD below the expected range for age (8). Evidence suggests bone loss is most pronounced in women with anorexia nervosa (26); while exercise-induced amenorrhea accompanied by weight loss confers particularly high fracture risk (27), despite the bone-protective effects of weight bearing exercise (28). Women with FHA who develop low BMD below the expected range for age face elevated risk of stress fractures, which occur in up to 30% of ballet dancers and other high-exercise populations (19, 29). Beyond low bone mass, chronic low-energy state suppresses bone formation and favors resorption, impairing repair of stress-related injuries (14). This uncoupling of bone remodeling, driven by the combined endocrine disturbances of FHA along with other behavioral components, underlies the disproportionate skeletal vulnerability in this population.

Remarkably, in the present study, FHA participants with prior COC use demonstrated lumbar spine BMD similar to eumenorrheic controls and significantly higher than recently menopausal women. These findings suggest a possible protective skeletal effect of COCs in FHA, although evidence remains mixed. A recent systematic review found that while two randomized controlled trials demonstrated BMD improvements with COC use (30, 31), several others reported no significant benefits (32). A network meta-analysis further found that the COC conferred no significant benefit for lumbar spine, femoral neck, or total hip BMD compared to control, whereas transdermal hormone replacement therapy was superior to control for both lumbar spine and femoral neck BMD (33). These differences may be explained in part by pharmacologic and exposure-related factors, including suppression of insulin-like growth factor I (IGF-I) with oral contraceptives, which may offset estrogen-mediated skeletal benefits, as well as the cyclic nature of COC administration (typically 21 days of active hormone exposure per 28-day cycle), resulting in intermittent rather than continuous estrogen exposure compared with the steady delivery achieved with transdermal estradiol therapy (22). To date, direct comparisons between continuous COC regimens and transdermal estrogen on bone outcomes remain limited.

It is important to note that hormonal therapy alone does not address the root cause of FHA. Management should prioritize nutritional and lifestyle interventions, with hormone therapy used adjunctively. The Endocrine Society’s clinical guidelines recommend against using COCs solely to restore menses or improve BMD in FHA due to limited benefit and the risk of masking spontaneous menstrual recovery (26). Instead, short-term transdermal estradiol with cyclic progestin is advocated after nutritional and lifestyle improvements, as addressing energy deficits alongside hormonal therapy offers the most effective approach to skeletal preservation in FHA (22).

Collectively, these findings reinforce that FHA is not a benign or transient condition. The associated skeletal and metabolic disturbances warrant early diagnosis, prompt lifestyle intervention, and multidisciplinary treatment. Restoration of menses alone may be insufficient to fully reverse bone loss (15, 19, 22). Given that up to 40% of adult peak bone mass accrues during adolescence and early adulthood, timely intervention is essential to reduce future fracture risk and preserve long-term musculoskeletal health (34).

### 4.1 Strengths and limitations

A key strength of our study is the rigorous phenotyping of participants with FHA, including repeated confirmation of hypoestrogenemia, low gonadotropin levels, and a low LH: FSH ratio, while systematically excluding other causes of secondary amenorrhea. This careful selection strengthens the internal validity of our findings. Additionally, our study is among the first to directly compare BMD in young women with FHA to both eumenorrheic controls and recently menopausal women, highlighting the extent of bone loss in FHA relative to physiological menopause. We also examined the influence of prior COC use on lumbar spine BMD, which revealed values comparable to those of eumenorrheic controls, suggesting a possible protective effect that warrants further investigation.

Despite these strengths, our study is limited by a relatively small sample size, although it remains one of the larger cohorts with phenotypically confirmed FHA and direct DXA-based bone assessments. Furthermore, the cross-sectional study design precludes causal inference and limits our ability to evaluate temporal changes in bone mineral density or establish longitudinal relationships between FHA, COC use, and skeletal outcomes. Prospective longitudinal studies are needed to better define these associations over time. Although we observed differences in lumbar spine BMD based on prior COC use, we lacked detailed information on the duration, formulation, dose, and timing of initiation, which may influence skeletal outcomes. More research is needed to explore whether COC use confers lasting skeletal benefits, and how factors such as the duration of COC use, timing of initiation, and the specific etiology of FHA (e.g., stress-, exercise-, or weight-related) influence bone outcomes. Future studies should explore how these behavioral and neuroendocrine features interact to mitigate bone loss and reduce fracture risk.

Importantly, we did not include biochemical markers of bone metabolism, such as serum calcium, phosphorus, alkaline phosphatase, parathyroid hormone, 25-hydroxyvitamin D, or bone turnover markers (e.g., procollagen type 1 N-terminal propeptide [P1NP], osteocalcin, and C-terminal telopeptide of type I collagen [CTX]). In addition, information on vitamin D supplementation, including prior or current use, dosage, and duration, was not collected. As a result, we were unable to evaluate underlying mechanisms of bone turnover or account for vitamin D status as a potential confounder to the observed differences in BMD. Future prospective studies should incorporate biochemical markers of bone metabolism and detailed vitamin D assessment to better characterize skeletal health in women with FHA and distinguish between changes in bone formation and bone resorption, thereby providing greater insight into the mechanisms underlying bone loss in this population. Finally, expanding research to include more racially and ethnically diverse populations will be important for enhancing the generalizability of these findings.

## 5 Conclusion

In this study, young women with FHA had significantly lower lumbar spine BMD than eumenorrheic controls, with values comparable to recently menopausal women, despite being decades younger. This highlights the serious impact of FHA on bone health during peak bone-building years. Prior COC use appeared to mitigate bone loss in the spine, suggesting a possible protective effect. Further research is needed to clarify whether bone outcomes vary by FHA subtype and duration of COC use.

## Data Availability

The raw data supporting the conclusions of this article will be made available by the authors, without undue reservation.

## Supplementary material

Supplementary Figure 1 Correlations Among Bone Mineral Density, Reproductive Hormones, and Oral Contraceptive Exposure in Women with Functional Hypothalamic Amenorrhea. Values represent Spearman rank correlation coefficients (ρ) among women with FHA (n = 20). Color intensity reflects the strength and direction of the correlation, with pink indicating positive and purple indicating negative associations. BMD, bone mineral density; LH, luteinizing hormone; FSH, follicle-stimulating hormone; LMP, last menstrual period; COC, combined oral contraceptive. Coefficients involving the binary COC Use variable represent rank-based (point-biserial) associations. *p<0.05; **p<0.01; **p<0.001.
